# Supplementary material for: FOXA1 loss drives basal/squamous de-differentiation of prostate cancer and induces an immunosuppressive tumor microenvironment
Source: Nat Commun. 2026 Mar 28;17:4572. doi: 10.1038/s41467-026-71121-8 (PMC13195071; doi:10.1038/s41467-026-71121-8)
Supplement: Supplementary file 1 — Supplementary Information [file 41467_2026_71121_MOESM1_ESM.pdf]

Supplemental Information

FOXA1 loss drives basal/squamous de-differentiation of prostate cancer and induces an immunosuppressive tumor microenvironment

Lourdes Brea, Hongshun Shi, Viriya Keo, Jing Huang, Liu Peng, Qi Chu, Wanqing Xie, Yinghua Xie, Sambhavi Senthil, Matthew T Breneman, Jie Fan, Ping Xie, Xiaodong Lu, David J. Degraff, Sarki A Abdulkadir, Ximing Yang, David Kosoff, Jonathan C Zhao, Bin Zhang, Jian Hu, Jindan Yu

The PDF file includes:

Figures S1 to S7

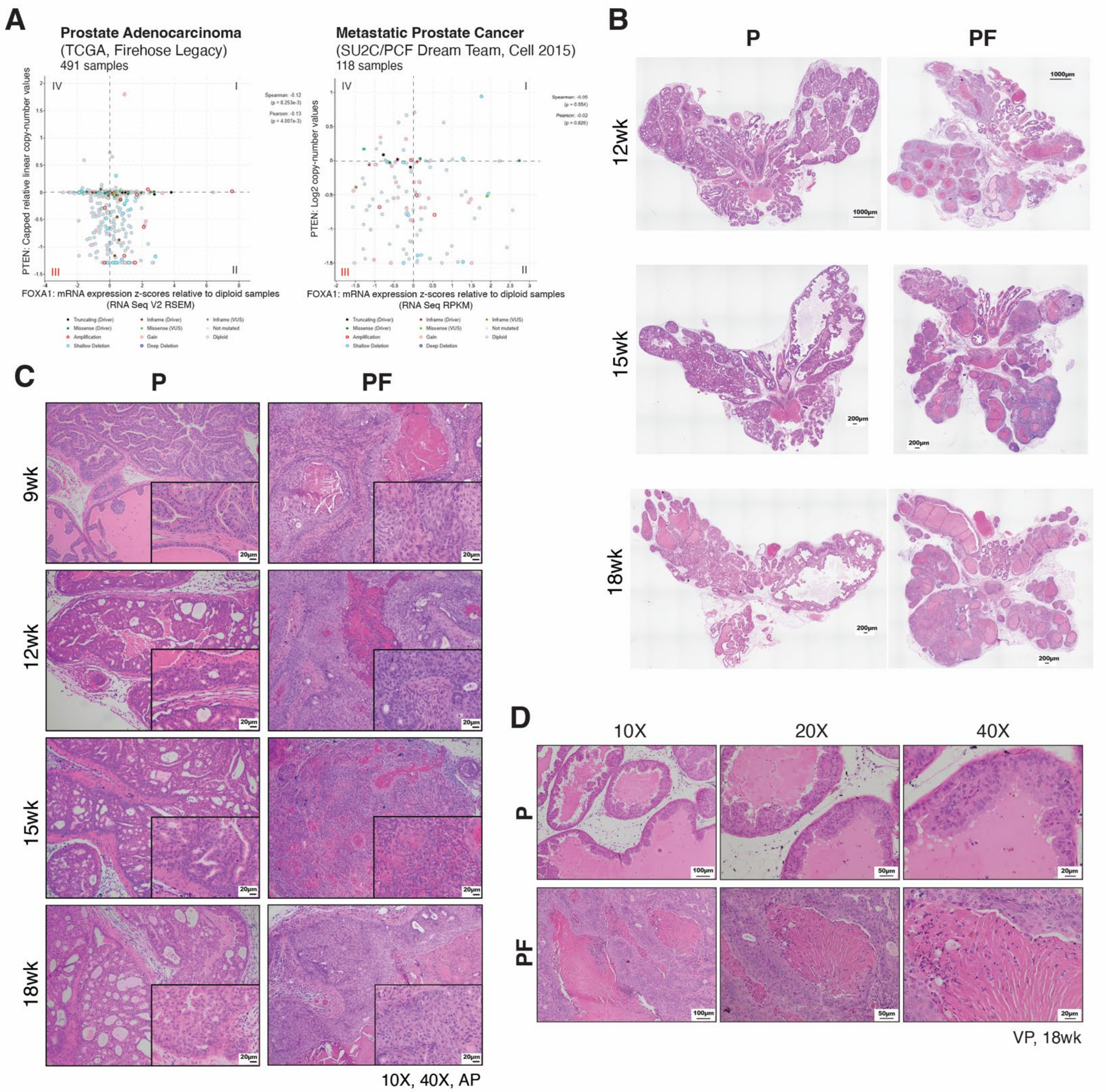

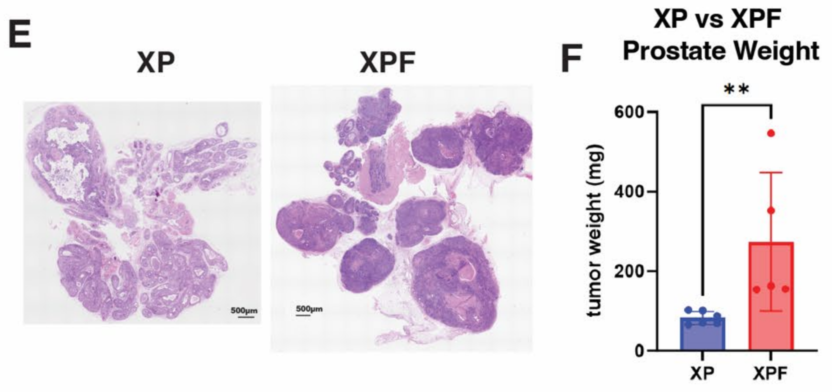

G

Sample Type	Total Mice Evaluated	Mice with Metastasis	Metastasis Incidence (%)
PF (intact)	9	0	0%
P (intact)	7	0	0%
XPF (castrated)	5	2	40%
XP (castrated)	5	0	0%

Figure S1. Effect of *Foxa1* deletion on mouse prostate tumor histopathology

A. cBioPortal correlation between PTEN copy number and FOXA1 mRNA expression relative to diploid samples in primary PCa TCGA Firehouse Legacy[1] (left, n = 491) and metastatic PCa SU2C/PCF Dream Team[2] (right, n = 118) patient samples. (n = number of patient samples with both mRNA and copy number data in each study.)
B. Whole tissue H&E images of P and PF prostates showing altered tissue architecture upon *Foxa1* loss. Similar histological findings were observed in ≥8 tumors from independent mice per genotype, with at least two tumors analyzed per time point.
C. Representative H&E staining of prostate tissues (AP lobe, 10X, 40X) from 9, 12, 15, and 18wk-old P and PF mice. Similar histological findings were observed in ≥8 tumors from independent mice per genotype, with at least two tumors analyzed per time point.
D. Representative H&E images of P18 vs PF18 prostate tumors demonstrating gained squamous differentiation and keratinization in PF mice (VP lobe, 10X, 20X, and 40X magnification). Similar results were observed in ≥8 tumors (from independent mice) per genotype.
E. Whole tissue images of castrated XP vs XPF prostate tissues demonstrating altered tissue architecture upon Foxa1 loss. Similar histological findings were observed across all analyzed mice (XP, n = 6; XPF, n = 5).
F. Prostate weights from XP and XPF mice castrated at 12–18 weeks of age and collected 3-12 months later. Data presented as the mean values of biological replicates (XP, n = 6; XPF, n = 5) ± SD, with individual points shown. Statistical significance was assessed using a two-sided Mann-Whitney U test ($p = 0.0043$). Source data are provided in the Source Data file.
G. Summary of number of mice identified to exhibit histopathological evidence of metastasis to lymph node and/or lung tissue. Source data are provided in the Source Data file.

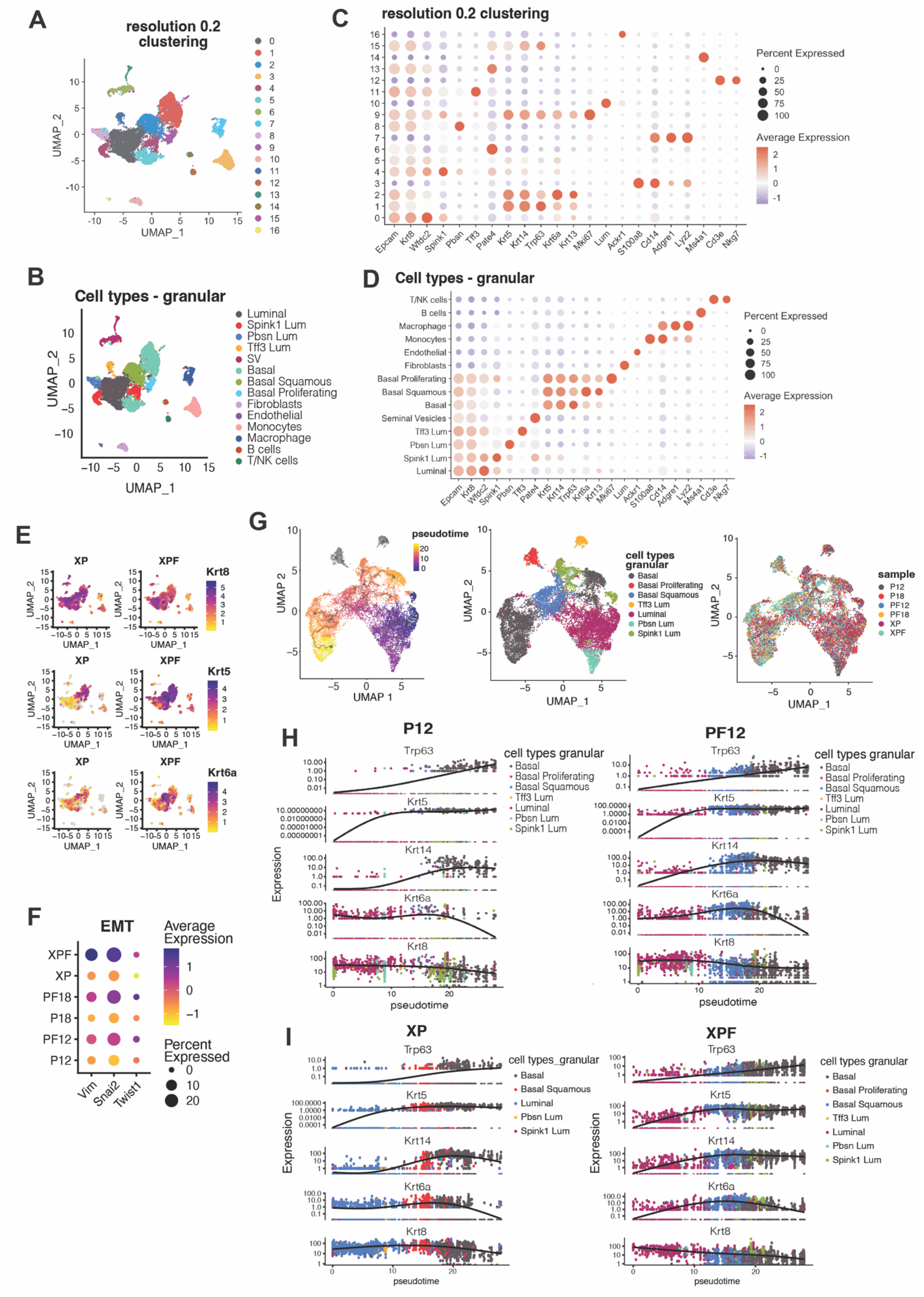
A
resolution 0.2 clustering
B
Cell types - granular
Luminal
Spink1 Lum
Pbsn Lum
Tff3 Lum
SV
Basal
Basal Squamous
Basal Proliferating
Fibroblasts
Endothelial
Monocytes
Macrophage
B cells
T/NK cells
C
resolution 0.2 clustering
Percent Expressed
Average Expression
D
Cell types - granular
E
XP
XPF
Krt8
Krt5
Krt6a
F
EMT
Average Expression
Percent Expressed
Vim
Snai2
Twist1
G
pseudotime
cell types granular
sample
H
P12
PF12
Trp63
Krt5
Krt14
Krt6a
Krt8
Expression
pseudotime
I
XP
XPF

Figure S2. Single-cell transcriptomics analysis of mouse prostate tumors

A. UMAP of integrated P12 (n = 2263 cells), PF12 (n = 3169 cells), P18 (n = 2738 cells), PF18 (n = 3584 cells), XP (n = 3590 cells), and XPF (n = 3518 cells) scRNA-seq samples colored by clusters (0-16) identified at resolution 0.2. The dataset includes one mouse prostate tumor analyzed per condition.
B. UMAP of integrated P12, PF12, P18, PF18, XP, and XPF scRNA-seq samples colored by granular cell types.
C. Dotplot demonstrating average expression of major cell type marker genes across resolution 0.2 clusters. Clusters 6 and 13 were combined into the seminal vesicle cell type in (B) due to their shared high expression of Pate4. Clusters 1, 5, and 15 were combined into the basal population in (B) based on their shared expression of key basal marker genes, though cluster 5 exhibits weaker expression of basal genes and may represent an intermediate luminal/basal state.
D. Dotplot demonstrating average expression of major cell type marker genes across granular cell types.
E. Feature plots depicting *Krt8* (luminal), *Krt5* (basal), and *Krt6a* (squamous) gene expression on the integrated UMAP for XP and XPF conditions.
F. Dot plot depicting the average expression and percent expression of EMT genes *Vim, Snai2*, and *Twist1* within epithelial cell populations in P vs PF tumors.
G. UMAP visualization of Monocle3 joint pseudo time trajectory analysis within epithelial population of integrated samples (P12, n = 1863 cells; PF12, n = 2407 cells; P18, n = 2252 cells; PF18, n = 2445 cells; XP, n = 2753 cells; XPF, n = 3195 cells). Upper UMAP depicting pseudotime trajectory. Middle UMAP depicting epithelial granular subtypes. Right UMAP depicting pseudotime trajectory. Lower UMAP depicting sample type.
H. Trajectory pseudotime analysis of epithelial cells within P12 and PF12 samples, performed using Monocle3. Luminal (*Krt8*), basal (*Krt5, Trp63, Krt14*), and squamous (*Krt6a*) gene expression are plotted across the joint pseudotime trajectory.
I. Trajectory pseudotime analysis of epithelial cells within XP and XPF samples, performed using Monocle3. Luminal (*Krt8*), basal (*Krt5, Trp63, Krt14*), and squamous (*Krt6a*) gene expression are plotted across the joint pseudotime trajectory.

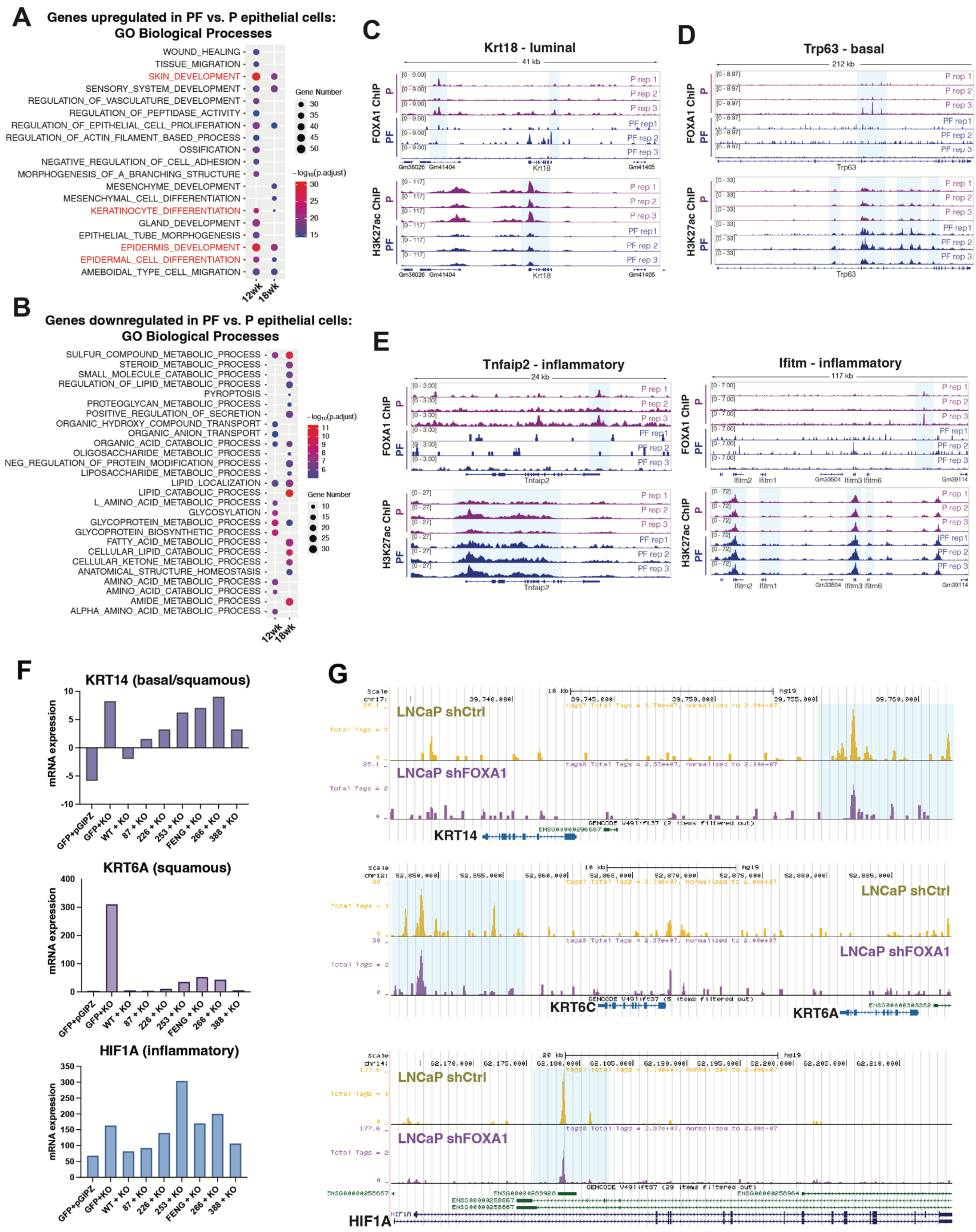

Figure S3. FOXA1 transcriptional regulation of lineage and inflammatory genes

A. GO analysis was performed on genes upregulated (log_2 fold change > 1) in PF vs. P epithelial cells from the scRNA-seq dataset across the 12wk (P12, n = 1863 cells; PF12, n = 2407 cells) and 18wk (P18, n = 2252 cells; PF18, n = 2445 cells) timepoints to assess enrichment of MSigDB GO biological processes (BP) gene sets. Pathways associated with squamous differentiation indicated in red. Gene set overlap significance was assessed using a hypergeometric test with FDR correction.
B. GO analysis of PF vs. P epithelial cells downregulated genes (log_2 fold change < -1) at 12wk and 18wk timepoints to assess enrichment of MSigDB GO biological processes (BP) gene sets. Gene set overlap significance was assessed using a hypergeometric test with FDR correction.
C. Genome browser tracks of FOXA1 and H3K27ac ChIP-seq intensity around luminal (Krt18) gene (n = 3 independent mouse prostate tumors for both P and PF genotypes).
D. Genome browser tracks of FOXA1 and H3K27ac ChIP-seq intensity around basal (Trp63) gene.
E. Genome browser tracks of FOXA1 and H3K27ac ChIP-seq intensity around inflammatory (Tnfaip2 and Ifitm2/3/6) genes.
F. LNCaP cells were transduced to KD FOXA1 and rescued with WT or mutant FOXA1 constructs, and RNA level was analyzed by microarray (one biological replicate per cell line condition). mRNA expression level for KRT14, KRT6A, and HIF1A are shown (GSE128882).[3]
G. Genome browser tracks at KRT14, KRT6A/C, and HIF1A for FOXA1 ChIP-seq in shCtrl vs shFOXA1 LNCaP cells (one biological replicate per cell line condition, GSE55007).[4]

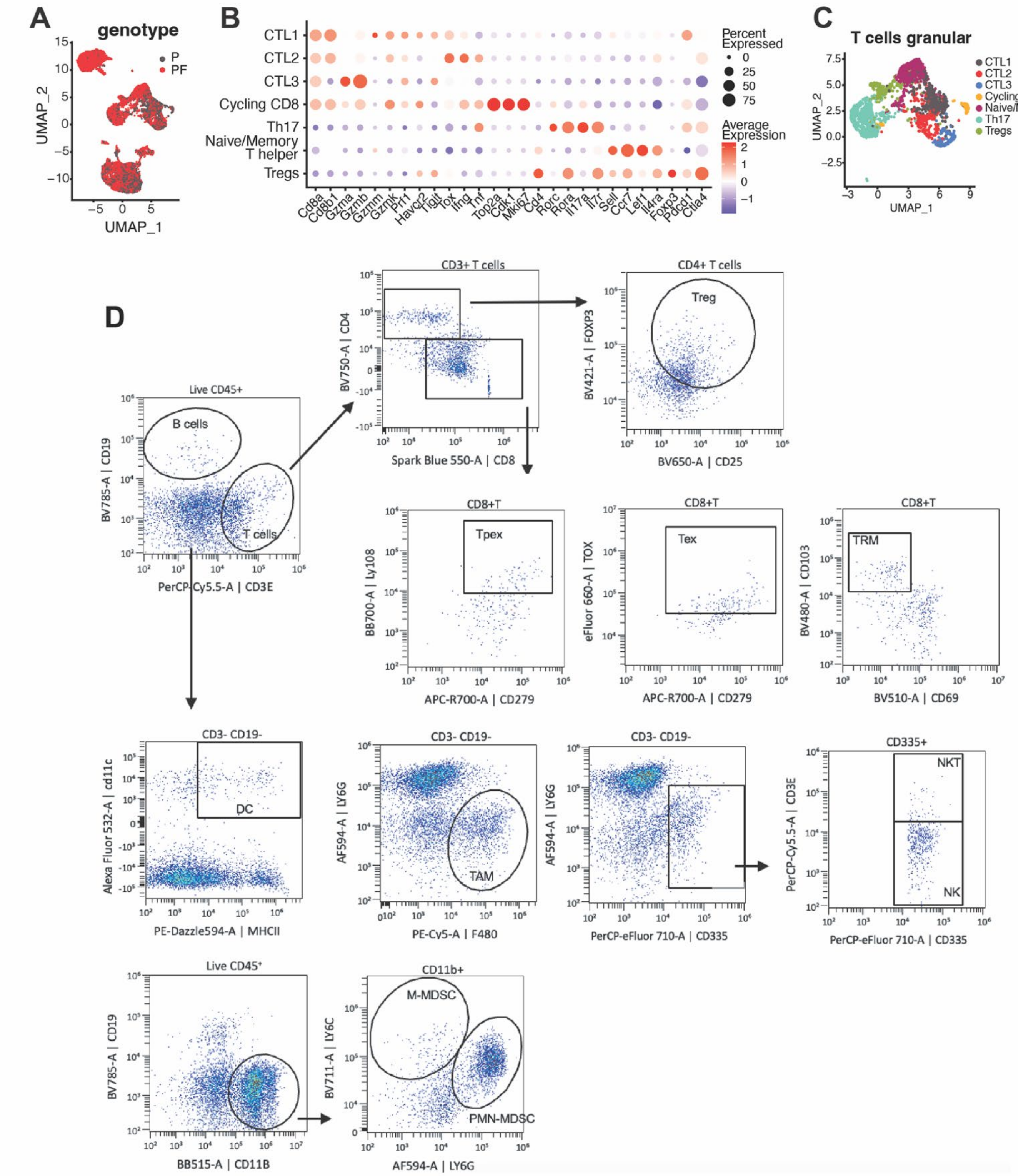

Figure S4. Single-cell transcriptomics and spectral flow cytometry analysis of mouse PCa immune infiltrates

A. UMAP for PIP-seq scRNA-seq samples colored by sample genotype. CD45+ cells from one pair of P (n = 3564 cells) vs PF (n = 8991 cells) age-matched 15wk mouse prostate tumors were analyzed by PIP-seq.
B. Expression of T cell subtype markers across different T cell subsets in PIP-seq samples.
C. UMAP of granular T cell subpopulations in PIP-seq samples (P15, n = 1357 T cells; PF15, n = 3112 T cells).
D. Example flow gating strategy for Cytek spectral flow cytometry analysis, corresponding to Figure 4F & I.

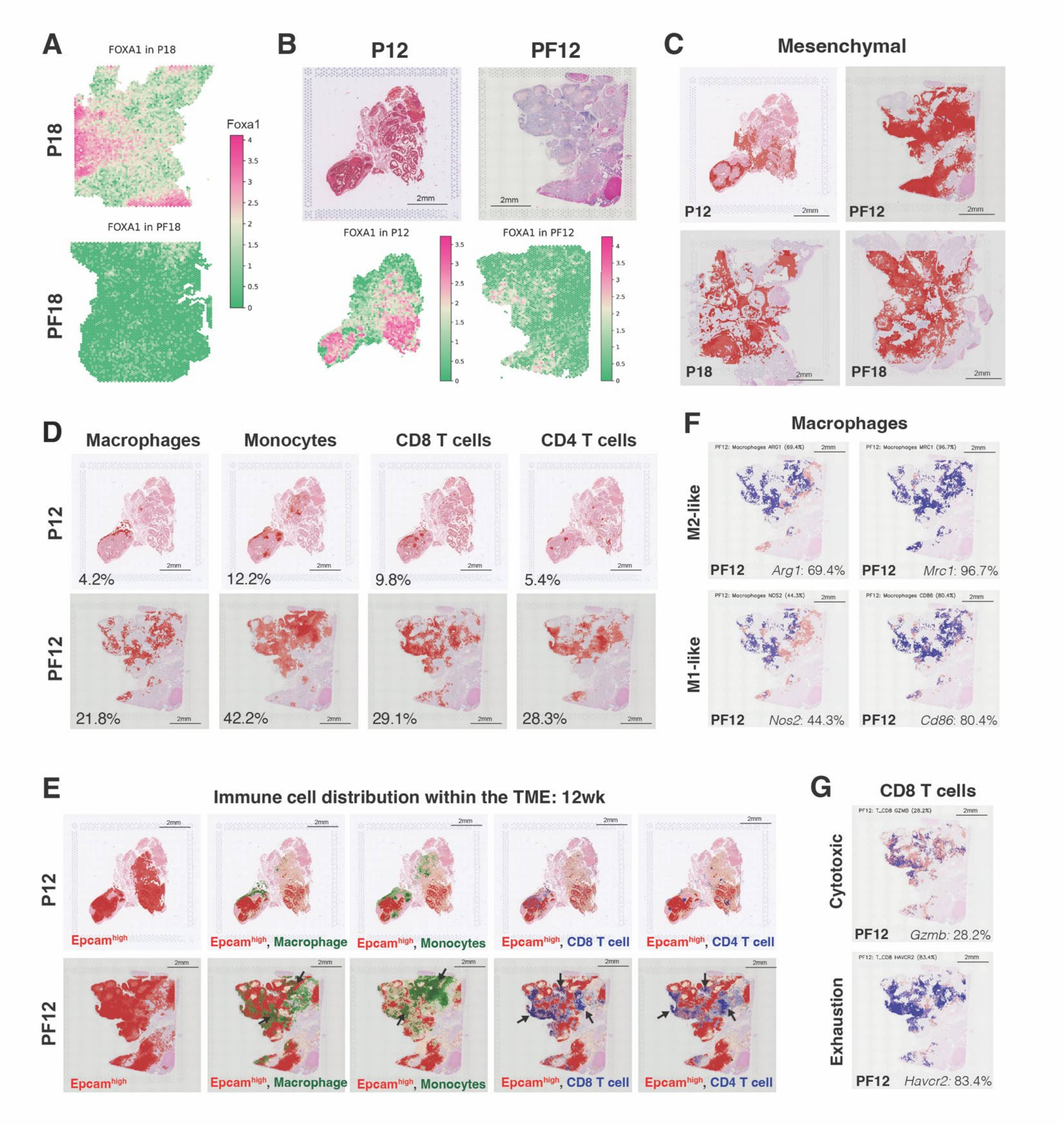

Figure S5. Spatial transcriptomics analysis of the mouse prostate tumor immune landscape

A. *Foxa1* expression in P18 vs. PF18 prostate tissue sections analyzed by 10x Genomics Visium.
B. H&E staining and *Foxa1* gene expression in P12 vs. PF12 Visium spatial transcriptomics samples.
C. TESLA analysis of mesenchymal/stromal populations (*Vim, Col1a1, Col3a1, Col6a1, Col6a2, Dcn, Tagln*) in P12, PF12, P18, and PF18 tissue sections profiled by Visium.
D. TESLA annotation of major immune groups of interest, macrophages, monocytes, CD4 T cells, and CD8 T cells, in P12 vs. PF12 Visium tissue sections. Darker red color indicates greater relative abundance of the indicated immune population. Percentages depicted represent the percent of total captured area classified as enriched for the indicated cell type

E. Epithelial tumor regions (*Epcam*^{high}) depicted in red were overlaid with macrophages (green), monocytes (green), CD4 T cells (blue), or CD8 T cells (blue) to visualize immune cell distribution within the tumor microenvironment in P12 and PF12 samples.
F. TESLA analysis of the percent of macrophage population in PF12 sample enriched for M2-like gene expression (*Arg1*+ 69.4%, *Mrc1*+ 96.7%) as compared to M1-like genes (*Nos2*+ 44.3%, *Cd86*+ 80.4%). (Light red indicates original identified macrophage population, blue indicates proportion of macrophage population enriched for indicated marker gene expression.)
G. TESLA analysis of the percent of CD8 T cell population identified in PF12 sample enriched for exhaustion marker (*Havcr2*+ 83.4%) and cytotoxic marker (*Gzmb*+ 28.2%) gene expression. (Light red indicates original identified parent population, blue indicates sub-population enriched for indicated marker gene expression).

(A-G) One mouse prostate tumor per genotype was analyzed by Visium at each timepoint (12 and 18 weeks; P12, PF12, P18, PF18), with similar trends observed across timepoints.

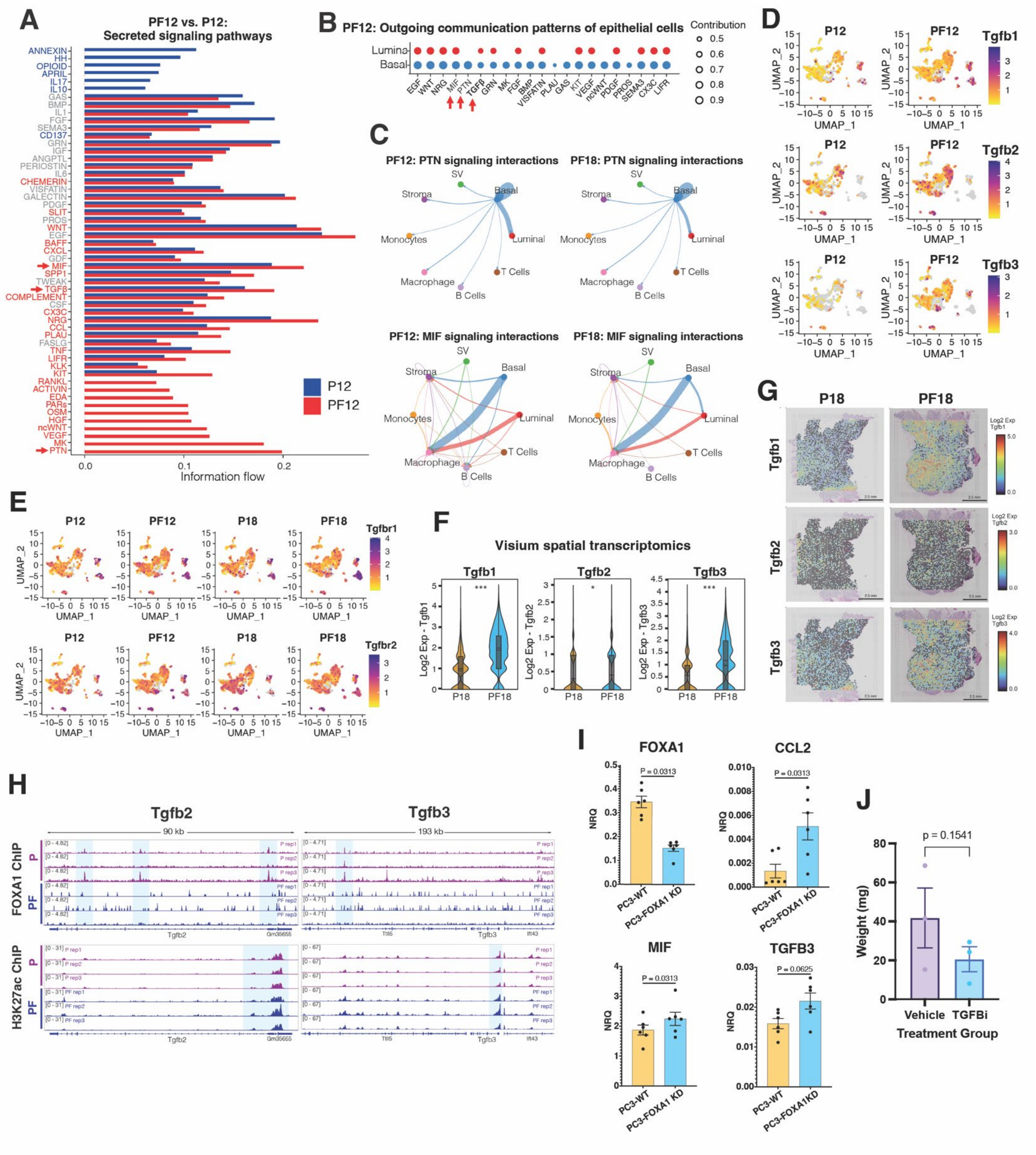

Figure S6. FOXA1 regulation of cell-cell communication and cytokine signaling in the mouse prostate TIME

A. Overall information flow for secreted signaling pathways. Pathways colored red are enriched in PF12, and those in blue are enriched in P12.
B. Dot plot showing major outgoing communication patters of epithelial populations in PF12 condition.

C. Circle plot depicting inferred source-target interactions for additional pathways of interest (PTN, MIF) identified as enriched in the PF condition and an outgoing communication pathway of basal and/or luminal cells.
D. *Tgfb3, Tgfb2,* and *Tgfb1* gene expression projected onto integrated UMAP for P12 (n = 2263 cells) and PF12 (n = 3169 cells) samples.
E. *Tgfbr1* and *Tgfbr2* expression projected onto integrated UMAP split by P12 (n = 2263 cells), PF12 (n = 3169 cells), P18 (n = 2738 cells), and PF18 (n = 3584 cells) conditions.
F. Quantification of *Tgfb1* (p = 1.65e-19), *Tgfb2* (p = 2.98e-2), and *Tgfb3* (p = 8.59e-14) cytokine expression in P18 vs PF18 spatial transcriptomics data. Differential gene expression was assessed across n = 3465 barcoded spots for P18 and n = 3903 barcoded spots for PF18 using Loupe Browser (10x Genomics) with a two-sided Wilcoxon rank-sum test with Benjamini–Hochberg correction for multiple testing. (***p-value < 0.001, **p-value < 0.01, *p-value < 0.05.)
G. Spatial transcriptomics maps demonstrating *Tgfb1, Tgfb2,* and *Tgfb3* cytokine expression across one age-matched pair of P18 and PF18 tissue sections.
H. IGV browser view of FOXA1 ChIP-seq occupancy at/near *Tgfb2 and Tgfb3* genes.
I. mRNA expression level of FOXA1, CCL2, MIF, and TGFB in PC3-WT vs PC3-FOXA1 KD cells co-cultured with patient-derived MDMs on a 3D biomatrix in Stacks. Data are presented as mean normalized relative quantity (NRQ) ± SEM, with individual points shown. Statistical significance was determined using a two-sided Wilcoxon matched-pairs signed-rank test (n = 6 biological replicates/patient samples). Source data are provided in the Source Data file.
J. Final tumor weight of PF derived allograft tumors treated with 75 mg/kg LY2157299 (TGFBi) twice a day for 3 weeks. Statistical significance was evaluated using a paired two-tailed t-test. Data presented as mean of 3 biological replicates (n = 3 mice) per treatment group ± SEM, with individual points shown. Source data are provided in the Source Data file.

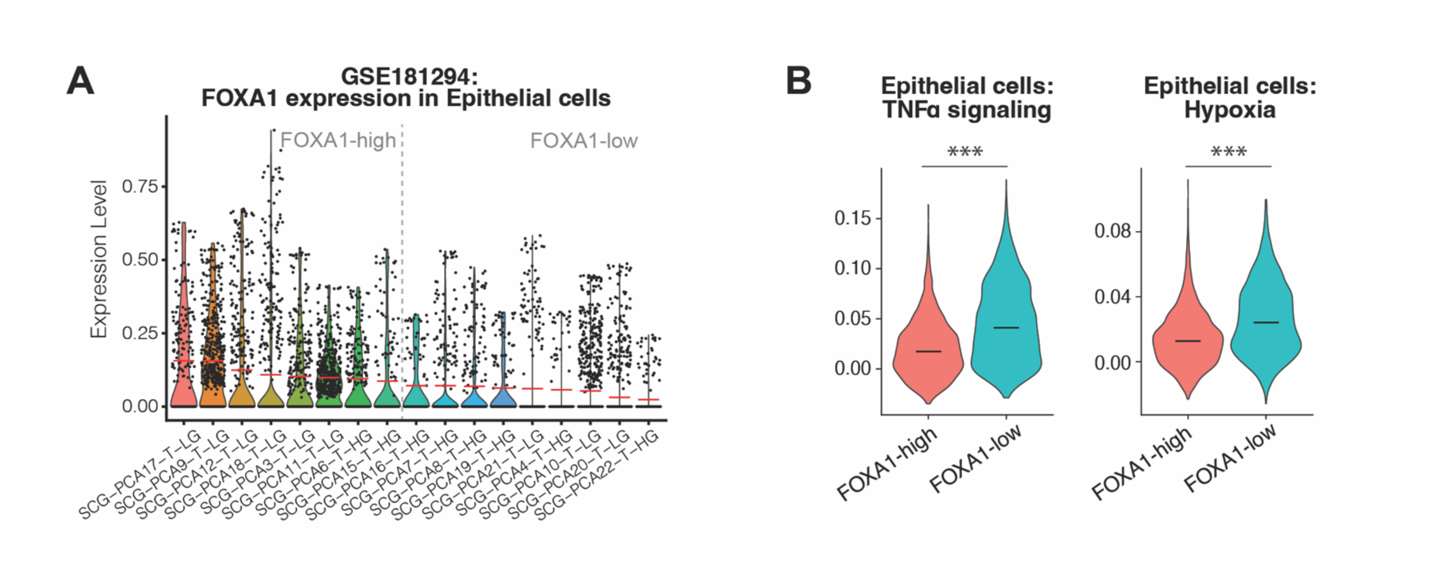

Figure S7. Transcriptomics analysis of the human PCa TIME

A. Violin plots of *FOXA1* expression within epithelial populations in PCa tumor samples from GSE181294,[5] with patients sorted from high to low *FOXA1* expression. Red horizontal bar indicates mean. Threshold for FOXA1-high vs low groups visualized by the gray dotted line.
B. Expression of Hallmark TNFα signaling ($p = 2.01e-146$) and Hypoxia ($p = 1.46e-106$) pathway genes within epithelial populations in *FOXA1*-low ($n = 3687$ epithelial cells) vs. *FOXA1*-high ($n = 3005$ epithelial cells) patients. Horizontal black bar indicates mean. Statistical significance determined using ggbetweenstats, Mann-Whitney nonparametric test.

References

1 The Molecular Taxonomy of Primary Prostate Cancer. *Cell* **163**, 1011-1025 (2015). https://doi.org/10.1016/j.cell.2015.10.025

2 Robinson, D. *et al.* Integrative Clinical Genomics of Advanced Prostate Cancer. *Cell* **161**, 1215-1228 (2015). https://doi.org/https://doi.org/10.1016/j.cell.2015.05.001

3 Xu, B. *et al.* Altered chromatin recruitment by FOXA1 mutations promotes androgen independence and prostate cancer progression. *Cell Res* **29**, 773-775 (2019). https://doi.org/10.1038/s41422-019-0204-1

4 Jin, H. J., Zhao, J. C., Wu, L., Kim, J. & Yu, J. Cooperativity and equilibrium with FOXA1 define the androgen receptor transcriptional program. *Nat Commun* **5**, 3972 (2014). https://doi.org/10.1038/ncomms4972

5 Hirz, T. *et al.* Dissecting the immune suppressive human prostate tumor microenvironment via integrated single-cell and spatial transcriptomic analyses. *Nature Communications* **14**, 663 (2023). https://doi.org/10.1038/s41467-023-36325-2
