# Supplementary material for: FOXA1 loss drives basal/squamous de-differentiation of prostate cancer and induces an immunosuppressive tumor microenvironment
Source: Nat Commun. 2026 Mar 28;17:4572. doi: 10.1038/s41467-026-71121-8 (PMC13195071; doi:10.1038/s41467-026-71121-8)
Supplement: Supplementary file 2 — Description of Additional Supplementary Files [file 41467_2026_71121_MOESM2_ESM.pdf]

Description of Additional Supplementary Files

Supplementary Data 1. Macrophage and T cell signature gene sets.

Supplementary Data 2. Cell type marker genes used for TESLA analyses.

Supplementary Data 3. CyTEK spectral flow cytometry antibodies.

Supplementary Data 4. Oligonucleotides used in this study.
