# Supplementary material for: FOXA1 loss drives basal/squamous de-differentiation of prostate cancer and induces an immunosuppressive tumor microenvironment
Source: Nat Commun. 2026 Mar 28;17:4572. doi: 10.1038/s41467-026-71121-8 (PMC13195071; doi:10.1038/s41467-026-71121-8)
Supplement: Supplementary file 4 — Reporting Summary [file 41467_2026_71121_MOESM4_ESM.pdf]

nature portfolio

Corresponding author(s): Jindan Yu

Last updated by author(s): February 21, 2026

Reporting Summary

Nature Portfolio wishes to improve the reproducibility of the work that we publish. This form provides structure for consistency and transparency in reporting. For further information on Nature Portfolio policies, see our Editorial Policies and the Editorial Policy Checklist.

Statistics

For all statistical analyses, confirm that the following items are present in the figure legend, table legend, main text, or Methods section.

n/a	Confirmed	
☐	☒	The exact sample size (*n*) for each experimental group/condition, given as a discrete number and unit of measurement
☐	☒	A statement on whether measurements were taken from distinct samples or whether the same sample was measured repeatedly
☐	☒	The statistical test(s) used AND whether they are one- or two-sided<br>*Only common tests should be described solely by name; describe more complex techniques in the Methods section.*
☐	☒	A description of all covariates tested
☐	☒	A description of any assumptions or corrections, such as tests of normality and adjustment for multiple comparisons
☐	☒	A full description of the statistical parameters including central tendency (e.g. means) or other basic estimates (e.g. regression coefficient) AND variation (e.g. standard deviation) or associated estimates of uncertainty (e.g. confidence intervals)
☐	☒	For null hypothesis testing, the test statistic (e.g. *F*, *t*, *r*) with confidence intervals, effect sizes, degrees of freedom and *P* value noted<br>*Give P values as exact values whenever suitable.*
☒	☐	For Bayesian analysis, information on the choice of priors and Markov chain Monte Carlo settings
☒	☐	For hierarchical and complex designs, identification of the appropriate level for tests and full reporting of outcomes
☐	☒	Estimates of effect sizes (e.g. Cohen's *d*, Pearson's *r*), indicating how they were calculated

Our web collection on statistics for biologists contains articles on many of the points above.

Software and code

Policy information about availability of computer code

Data collection	scRNA-seq: Chromium Next GEM Single Cell 3'3' Kit v3.1<br>PIP-seq: PIPseq T20 3% Single Cell RNA Kit v3.0<br>Spatial Transcriptomics: Visium Spatial for FFPE Gene Expression Kit, Mouse Transcriptome<br>Flow cytometry:CyTEK AURORA spectral flow cytometry<br>NGS:HiSEQ4000 platform with Illumina RTA 2.7.7; NOVASEQ6000,S4 Reagent Kit v1.5; NOVASEQ X PLUS 10B Reagent Kit (Cat# 20085594).
Data analysis	scRNA-seq analysis:<br>Raw sequencing data were preprocessed and aligned to hg38 using Cell Ranger ARC (6.1.2).<br>Low-quality cells with low UMI counts and high mitochondrial ratios were filtered out in Seurat/4.3.0<br>The individual samples were normalized using SCTransform and integrated with castrated mice samples using the Seurat V3

Monocle3 (0.2.3.0) was used to learn the trajectory graph and calculate the pseudotime
Dittoseq v1.20.1 was utilized to visualize cell type proportions
Seurat FindMarkers() function and the MAST package were used for differentially expressed (DE) genes analysis
Seurat AddModuleScore() function was utilized to evaluate module scores for gene expression programs
CellChat analysis of 10x scRNA-seq data:
Cell-cell interactions were evaluated using the CellChat v2 R package
Spatial transcriptomics analysis:
TESLA, a machine learning method, was utilized to annotate cell type distribution of Visium spatial transcriptomics data
Flow cytometry and CyTEK analysis:
Data analysis was performed using either the OMIQ web application (https://www.omiq.ai/) or the FlowJo application, according to previously described methods (PMID: 35671108; PMID: 38530357)
The code for next-generation sequencing analysis performed in this paper has been uploaded to https://github.com/JYULAB/FOXA1_immune_project.
ChIP-seq analysis:
Basecalls were performed using Bowtie2 v2.5.1 for Novaseq output
ChIP-seq reads were trimmed from 3' end untile the final base has a quality score > 30, using Trimmomatic v0.39, discarding reads left with < 36 bp
ChIP-seq reads were aligned to the mm10 genome using Bowtie2 v2.5.1.
Duplicate reads were identified and removed using Picard v3.0.0.
Peaks were calling using HOMER v4.11 with default settings.
bigWig files were generating using deepTools v3.5.4 with binsize = 10bp

For manuscripts utilizing custom algorithms or software that are central to the research but not yet described in published literature, software must be made available to editors and reviewers. We strongly encourage code deposition in a community repository (e.g. GitHub). See the Nature Portfolio guidelines for submitting code & software for further information.

Data

Policy information about availability of data

All manuscripts must include a data availability statement. This statement should provide the following information, where applicable:

- Accession codes, unique identifiers, or web links for publicly available datasets
- A description of any restrictions on data availability
- For clinical datasets or third party data, please ensure that the statement adheres to our policy

Data Availability
All scRNA-seq, ChIP-seq, and Visium spatial transcriptomics data generated for this study have been deposited in the Gene Expression Omnibus (GSE282726) at https://www.ncbi.nlm.nih.gov/geo/query/acc.cgi?acc=GSE282726. Previously published microarray data for LNCaP cells with FOXA1 KD and rescue with WT or mutant FOXA1 constructs is deposited in GSE12888213 (https://www.ncbi.nlm.nih.gov/geo/query/acc.cgi?acc=GSE128882). Previously published data for FOXA1 ChIP-seq in LNCaP cells is deposited in GSE550076 (https://www.ncbi.nlm.nih.gov/geo/query/acc.cgi?acc=GSE55007). Publicly available human PCa datasets analyzed in this study can be found at GSE18129421 (https://www.ncbi.nlm.nih.gov/geo/query/acc.cgi?acc=GSE181294) and GSE2103459 (https://www.ncbi.nlm.nih.gov/geo/query/acc.cgi?acc=GSE21034). With regards to biospecimen data related to the in vitro co-culture assays, the institutional biospecimen collection protocol does not allow unrestricted public access to the raw data to maintain protection of patient privacy. Therefore, data sharing requests must be submitted to the William S Middleton Memorial Veterans Hospital for review and approval. Source data are provided with this paper.

Research involving human participants, their data, or biological material

Policy information about studies with human participants or human data. See also policy information about sex, gender (identity/presentation), and sexual orientation and race, ethnicity and racism.

Reporting on sex and gender	N/A
Reporting on race, ethnicity, or other socially relevant groupings	N/A
Population characteristics	N/A
Recruitment	N/A
Ethics oversight	Ethics Statement<br>The study was conducted in compliance with the Declaration of Helsinki. Patients were enrolled under institutional IRB-approved biospecimen protocols (1202-1214 and 2020-0915).<br><br>Consent<br>Written informed consent was obtained from all participants before enrollment.

Note that full information on the approval of the study protocol must also be provided in the manuscript.

Field-specific reporting

Please select the one below that is the best fit for your research. If you are not sure, read the appropriate sections before making your selection.

☒ Life sciences ☐ Behavioural & social sciences ☐ Ecological, evolutionary & environmental sciences

For a reference copy of the document with all sections, see nature.com/documents/nr-reporting-summary-flat.pdf

Life sciences study design

All studies must disclose on these points even when the disclosure is negative.

Sample size	No statistical methods were utilized to predetermine the sample sizes. The sample sizes used in current study were similar to previous studies (PMID: 36332622,PMID: 34099734) and included in figures/figure legends.
Data exclusions	No data was excluded
Replication	For scRNA-seq experiments, samples at different timepoints (week12 and week18) from same genotype were used for 10x Genomics scRNAseq, the data from two timepoints were highly consistent. In addition, the data were well validated in independent assays including PIP-seq, Visium and CyTEK assays. For IHC and HE experiments, three or more tumors (from independent mice) were analyzed. For ChIP-seq experiments, ChIP-seq was performed in triplicate biological samples for age-matched P vs PF tumors.
Randomization	For in vivo drug treatment experiment, mice were randomly assigned to control and treatment groups.<br>Randomization is not applicable to other experiments involving genetically engineered mice.
Blinding	Investigators were not blinded to group allocation during data collection and analysis.

Reporting for specific materials, systems and methods

We require information from authors about some types of materials, experimental systems and methods used in many studies. Here, indicate whether each material, system or method listed is relevant to your study. If you are not sure if a list item applies to your research, read the appropriate section before selecting a response.

Materials & experimental systems

n/a	Involved in the study	
☐	☒	Antibodies
☐	☒	Eukaryotic cell lines
☒	☐	Palaeontology and archaeology
☐	☒	Animals and other organisms
☒	☐	Clinical data
☒	☐	Dual use research of concern
☒	☐	Plants

Methods

n/a	Involved in the study	
☐	☒	ChIP-seq
☐	☒	Flow cytometry
☒	☐	MRI-based neuroimaging

Antibodies

Antibodies used

Antibodies for IHC:
Recombinant Anti-FOXA1 antibody [EPR10881] ,ab170933, Rabbit (1:1000).
PTEN (D4.3) XP® Rabbit mAb , Cell Signaling Technology, #9188 (1:75)
Recombinant Anti-Androgen Receptor antibody [ER179(2)] , ab108341, Rabbit (1:1000)
Anti-p63 Antibody (D-9), Santa Cruz sc-25268 , Mouse(1:250)
Purified anti-Cytokeratin 8 Antibody , Biolegend, 904804, Mouse, (1:400)
pSmad2 (MilliporeSigma; AB3849; 1:1000)
F4/80 (CST 70076; 1:500)
CD4 (CST 25229; 1:100)
CD8 (CST-98941; 1:400).
Antibodies for ChIP-seq: FOXA1 ab23738 (5µg; Abcam) and H3K27ac CST 8173S (1:100)
Antibodies for sorting CD45+ tumor infiltrating immune cells:
Fc block TruStain FcX™ PLUS Antibody # 156603 (1:150)
Biolegend APC anti-mouse CD45 Antibody # 147707 (1:100)
Antibodies used for CyTEK analysis:
CD4-BV750 (GK1.5) 1:100 Biolegend 100467
CD8a-Spark Blue550 (53-6.7) 1:100 Biolegend 100780
CD45-BV570 (30-F11) 1:100 Biolegend 103135
CD3e-Percp5.5 (145-2C11) 1:100 Biolegend 100328
CD11b-BB515 (M1/70) 1:100 BD Horizon 564454
CD11c-AF532 (N418) 1:100 eBiosciences 58011482

	CD19-BV785 (6D5) 1:100 Biolegend 115543<br>CD25-BV650 (PC61) 1:50 Biolegend 102038<br>CD44-PECy5 (IM7) 1:50 Biolegend 103010<br>CD69-BV510 (H1.2F3) 1:50 Biolegend 104532<br>CD73-APC (TY/11.8) 1:50 Biolegend 127210<br>IFN-γ-PECy7 (XMG1.2) 1:50 Biolegend 505826<br>TNF-α-BV650 (MP6-XT22) 1:50 Biolegend 506333<br>F4/80-PECy5 (BM8) 1:100 Biolegend 123114<br>MHCII-PE/Dazzle™ 594 (M5/114.15.2) 1:100 Biolegend 121606<br>CD163-PE (S15049F) 1:100 Biolegend 156704<br>CD274-BV605 (10F.9G2) 1:100 Biolegend 124321<br>CD279-APC-R700 (J43) 1:50 BD Biosciences 565815<br>Ly-6C-BV711 (HK1.4) 1:100 Biolegend 128037<br>Ly-6G-AF594 (1A8) 1:100 Biolegend 127636<br>Tim3-APC (B8.2C12) 1:50 Biolegend 134008<br>CD335(NKp46)-PerCp-eFluor710 1:100 eBiosciences 46-3351-82<br>CX3CR1-APC/Fire750 (SA011F11) 1:100 Biolegend 149039<br>CD86-FITC (GL-1) 1:100 Biolegend 105006<br>FoxP3-BV421 (MF-14) 1:25 Biolegend 126419<br>Ly108-BB700 (13G3) 1:100 BD Biosciences 742272<br>Ki67-PacificBlue (16A8) 1:50 Biolegend 652422<br>Tox-eFluor660 (TXRX10) 1:100 eBiosciences 50650282<br>CD103-BV480 (M290) 1:50 BD Biosciences 566118<br>KLRG1-PECy7 (2F1/KLRG1) 1:50 Biolegend 138416<br>CD45-APC-Cy7 (30-F11) 1:100 Biolegend 103116<br>CD8-BUV737 (53-6.7) 1:100 BD Biosciences 612759<br>CD4-FITC (GK1.5) 1:100 Biolegend 100406<br>CD279 (PD1)-PE (29F.1A12) 1:50 Biolegend 135206<br>Zombie yellow 1:500 Biolegend 423103<br>Live/Dead fixable blue 1:500 ThermoFisher L23105
Validation	All antibodies were validated by manufacturers and previously used in several publications.<br>Anti-FOXA1 antibody [EPR10881] used for IHC assay was validated in our Foxa1 KO mice and PMID: 33785741<br>PTEN (D4.3) XP® Rabbit mAb used for IHC assay was validated in our Pten KO mice and PMID: 29057879<br>Recombinant Anti-Androgen Receptor antibody [ER179(2)] used for IHC assay was validated in PMID: 23817021<br>Anti-p63 Antibody (D-9) used for IHC assay was validated in PMID: 23620512<br>Purified anti-Cytokeratin 8 Antibody used for IHC assay was validated in PMID: 31390564<br>pSmad2 antibody for IHC was validated in PMID: 28806779<br>F4/80 (CST 70076; 1:500), CD4 (CST 25229; 1:100), CD8 (CST-98941; 1:400) are validated by the manufacturer for IHC<br>FOXA1 ab23738 (Abcam) antibody for ChIP-seq was validated in PMID: 24875621<br>H3K27ac CST 8173S antibody is validated by the manufacturer for ChIP-seq<br>Biolegend APC anti-mouse CD45 Antibody # 147707 used for FACS was broadly validated in literature<br>Antibodies used for Cytek flow analysis have been validated by the manufacturer

Eukaryotic cell lines

Policy information about cell lines and Sex and Gender in Research

Cell line source(s)	PC3 cell line was obtained from ATCC.<br>PBMCs were isolated from blood samples collected from male patient donors with prostate cancer after receiving written informed consent under a protocol approved by the Institutional Review Board at the William S Middleton Memorial Veteran's Hospital, Madison, WI in accordance with the Declaration of Helsinki.
Authentication	PC3 cell line was obtained from ATCC and periodically tested for potential mycoplasma contamination.
Mycoplasma contamination	PC3 cell line tested negative for mycoplasma.
Commonly misidentified lines<br>(See ICLAC register)	No commonly misidentified lines were used in this study

Animals and other research organisms

Policy information about studies involving animals; ARRIVE guidelines recommended for reporting animal research, and Sex and Gender in Research

Laboratory animals	Genetically engineered mice for this study were generated on a C57BL/6 background. Foxa1f/f mouse, generously shared by Dr. David Degraff. PbCre4:Ptenf/f and PbCre4:Ptenf/fFoxa1f/f mice were generated in the lab<br>NSG mice were used to expand PF tumor allografts, which were subsequently re-engrafted into NOD-SCID mice for in vivo TGFβ receptor I inhibitor treatment study.
Wild animals	Wild animals were not included in the study
Reporting on sex	Male and female mice were used for breeding. The findings in this study were only applicable to male mice.

Field-collected samples	No field-collected samples were used in this study
Ethics oversight	All mouse work was approved by the Institutional Animal Care and Use Committee (IACUC) at Northwestern University and Emory University in compliance with all relevant ethical regulations.

Note that full information on the approval of the study protocol must also be provided in the manuscript.

Plants

Seed stocks	N/A
Novel plant genotypes	N/A
Authentication	N/A

ChIP-seq

Data deposition

☒ Confirm that both raw and final processed data have been deposited in a public database such as GEO.

☒ Confirm that you have deposited or provided access to graph files (e.g. BED files) for the called peaks.

Data access links *May remain private before publication.*	All scRNA-seq, ChIP-seq, and Visium spatial transcriptomics data generated for this study have been deposited in the Gene Expression Omnibus (GSE282726) at https://www.ncbi.nlm.nih.gov/geo/query/acc.cgi?acc=GSE282726.
Files in database submission	*Provide a list of all files available in the database submission.*
Genome browser session (e.g. UCSC)	ChIP-seq data is being deposited in GSE282726 at https://www.ncbi.nlm.nih.gov/geo/query/acc.cgi?acc=GSE282726

Methodology

Replicates	FOXA1 and H3K27ac ChIP-seq was performed in biological triplicates for age-matched P vs. PF prostate tumors
Sequencing depth	paired-end (NoVaSeq X plus PE150)
Antibodies	FOXA1 ab23738 (Abcam) and H3K27ac CST 8173S<br>Drosophila H3 antibody (Active motif Cat# 61686) was also added per sample for spike-in control
Peak calling parameters	Peaks were calling using HOMER v4.11 with default settings.
Data quality	Duplicate reads were identified and removed by Picard (v3.0.0) and the adaptor reads removal process was performed with Trimmomatic V0.39. Reads were aligned against the Mus musculus reference genome mm10 and the D. melanogaster reference genome Dmel A4.10 using Bowtie2 (v2.5.1). Spike-in normalization was performed by counting D. melanogaster reads in each sample to calculate a spike-in normalization factor, and down-sampling was performed by samtools (v1.17) by random down-sampling to the smallest spike-in counts. HOMER (v4.11) was utilized for ChIP-seq peak calling with default cutoff.
Software	The code for the code for next-generation sequencing analysis performed in this paper is uploaded to https://github.com/JYULAB/FOXA1_immune_project.

Flow Cytometry

Plots

Confirm that:

☒ The axis labels state the marker and fluorochrome used (e.g. CD4-FITC).

☒ The axis scales are clearly visible. Include numbers along axes only for bottom left plot of group (a 'group' is an analysis of identical markers).

☒ All plots are contour plots with outliers or pseudocolor plots.

☒ A numerical value for number of cells or percentage (with statistics) is provided.

Methodology

Sample preparation	The single-cell suspensions underwent stimulation with phorbol 12-myristate 13-acetate (50 ng/ml), ionomycin (5 μg/ml), and brefeldin A (10 μg/ml) to induce IFNγ and TNFα production. To block non-specific antibody binding to Fc receptors on immune cells, the stimulated single-cell suspensions were incubated with anti-mouse CD16/32 antibody (2.4G2). Following this, live/dead dye and fluorophore-conjugated anti-mouse antibodies targeting cell surface markers were added. After PBS washing, cells were fixed with 1x Fixation/Permeabilization reagent (eBioscienceTM, 00-5223-56) for 20 minutes at room temperature. Subsequently, cells were washed twice with 1x Permeabilization/wash buffer (eBioscienceTM, 00-8333-56) and subjected to intracellular staining (ICS) with an antibody cocktail for 45 minutes at room temperature.
Instrument	Three-laser CyTEK AURORA spectral flow cytometry or LSRII flow cytometry
Software	OMIQ (https://www.omiq.ai/), FlowJo™ v10
Cell population abundance	Relative abundance of each gated population can be found in Fig 4F
Gating strategy	Gating strategy information can be found in Supplementary Fig. S4D

☒ Tick this box to confirm that a figure exemplifying the gating strategy is provided in the Supplementary Information.
